# The interferon type I signature towards prediction of non-response to rituximab in rheumatoid arthritis patients

**DOI:** 10.1186/ar3819

**Published:** 2012-04-27

**Authors:** Hennie G Raterman, Saskia Vosslamber, Sander de Ridder, Michael T Nurmohamed, Willem F Lems, Maarten Boers, Mark van de Wiel, Ben AC Dijkmans, Cornelis L Verweij, Alexandre E Voskuyl

**Affiliations:** 1 Department of Rheumatology, VU University medical center, de Boelelaan 1117, Amsterdam, 1081HV, the Netherlands; 2 Department of Pathology, VU University medical center, de Boelelaan 1118, Amsterdam, 1081HV, Amsterdam, the Netherlands; 3 Department of Rheumatology, Jan van Breemen Research Institute|Reade, Jan van Breemenstraat 2, Amsterdam, 1056AB, The Netherlands; 4 Department of Epidemiology and Biostatistics, VU University medical center, de Boelelaan 1117, Amsterdam, 1081HV, the Netherlands

## Abstract

**Introduction:**

B cell depletion therapy is efficacious in rheumatoid arthritis (RA) patients failing on tumor necrosis factor (TNF) blocking agents. However, approximately 40% to 50% of rituximab (RTX) treated RA patients have a poor response. We investigated whether baseline gene expression levels can discriminate between clinical non-responders and responders to RTX.

**Methods:**

In 14 consecutive RA patients starting on RTX (test cohort), gene expression profiling on whole peripheral blood RNA was performed by Illumina^® ^HumanHT beadchip microarrays. Supervised cluster analysis was used to identify genes expressed differentially at baseline between responders and non-responders based on both a difference in 28 joints disease activity score (ΔDAS28 < 1.2) and European League against Rheumatism (EULAR) response criteria after six months RTX. Genes of interest were measured by quantitative real-time PCR and tested for their predictive value using receiver operating characteristics (ROC) curves in an independent validation cohort (*n *= 26).

**Results:**

Genome-wide microarray analysis revealed a marked variation in the peripheral blood cells between RA patients before the start of RTX treatment. Here, we demonstrated that only a cluster consisting of interferon (IFN) type I network genes, represented by a set of IFN type I response genes (IRGs), that is, *LY6E, HERC5, IFI44L, ISG15, MxA, MxB, EPSTI1 *and *RSAD2*, was associated with ΔDAS28 and EULAR response outcome (*P *= 0.0074 and *P *= 0.0599, respectively). Based on the eight IRGs an IFN-score was calculated that reached an area under the curve (AUC) of 0.82 to separate non-responders from responders in an independent validation cohort of 26 patients using Receiver Operator Characteristics (ROC) curves analysis according to ΔDAS28 < 1.2 criteria. Advanced classifier analysis yielded a three IRG-set that reached an AUC of 87%. Comparable findings applied to EULAR non-response criteria.

**Conclusions:**

This study demonstrates clinical utility for the use of baseline IRG expression levels as a predictive biomarker for non-response to RTX in RA.

## Introduction

Rheumatoid arthritis (RA) is a systemic autoimmune disease characterized by chronic inflammation of the joints that may cause permanent cartilage and bone destruction. Currently, no curative treatment is available, and patients are subjected to a prolonged course of treatment. RA is marked by the presence of rheumatoid factor (RF) and/or anti-citrullinated protein autoantibodies (ACPA), which may precede the appearance of clinical symptoms of arthritis by many years [1,2]. Surface expressing RF B-cells may bind immune complexes and thereby serve a role as efficient antigen presenting cells that could lead to a break in T-cell tolerance against autoantigens [3]. In addition, an arthritogenic role for ACPA in experimental models of arthritis has been demonstrated [4,5]. Besides producers of auto-antibodies, B cells may contribute to disease pathogenesis through their role in antigen presentation, lymphoneogenesis and cytokine release [6]. Therefore, it was suggested that B-cells are essential players of the disturbed immune system, which fuelled interest in B-cells as drug target.

Rituximab (RTX) is a chimeric-human monoclonal antibody directed against the B cell marker CD20 that effectively depletes CD20-positive B cells. RTX is efficacious and safe in RA patients who are failing on TNF blocking agents [7-9]. Despite the effective depletion of circulating B cells in nearly all treated patients, clinical experience revealed that approximately 40% to 50% of RA patients do not respond to RTX [8,9]. Considering the progression of damage and the high costs of treatment with biologicals, identification of non-responders before start of treatment is highly desirable.

Clinical parameters such as baseline disability, number of previously used TNF blocking agents, and reason for ineffectiveness of anti-TNF treatment were found to be associated with non-response to RTX [10,11]. Whereas fluorescence activated cell sorter (FACS) studies revealed no association between B cell numbers at baseline and clinical outcome, highly sensitive FACS technology suggested that the failure for complete B cell depletion at six months was associated with a poor response [12]. Pooled data from ten European registries (CARRERA) demonstrated that seropositive patients achieved significantly greater reductions in 28 joint disease activity score (DAS28) at six months than seronegative patients [13]. Others reported associations between BAFF/BLyS levels, FcγRIII and IL-6 genotype, and Epstein-Barr virus genome in bone marrow and clinical outcome [10,14,15]. In addition, preliminary studies suggested an association between the expression level of transcripts in peripheral blood cells and clinical outcome [16,17]. Overall these findings have potential to provide a framework to select clinically relevant predictors but require validation and subsequent prognostic evaluation of clinical utility to warrant further development.

In the present study we focus on further analysis of transcript biomarkers in predicting response to RTX in order to determine the value of the proclaimed transcript markers and extend the transcript profile associated with response outcome. Key questions that we want to answer are: 1) Can previously reported transcript markers that are associated with clinical response be confirmed?; 2) What is the complexity of the transcript profile that is associated with responsiveness?; 3) Can we demonstrate the clinical utility of the transcript markers in an independent study? and 4) Can we optimize the transcript biomarker set to increase its predictive capacity? Therefore, we started whole genome transcript profiling of whole peripheral blood cells from patients prior to RTX treatment to study mRNA levels of all the genes in the genome simultaneously. Receiver Operating Characteristics (ROC)-curve analysis in an independent validation study was applied to demonstrate clinical utility and selection of the most optimal transcript biomarker set.

## Materials and methods

### Patient recruitment

Forty consecutive RA patients (14 for the test cohort and 26 for the validation cohort), according to the 1988 revised American College of Rheumatology criteria for the diagnosis of RA, attending the outpatient clinics of the VU University medical center and Jan van Breemen Research Institute|READE scheduled for RTX were followed prospectively [18]. Inclusion criteria for this study are according to the guidelines of the Dutch Society for Rheumatology for treatment with RTX, that is, active disease status (defined as DAS28 > 3.2 at treatment initiation) despite previous treatment with TNF-blocking agents, unless contraindicated in the opinion of the treating physician and previous treatment with methotrexate (MTX) and one other disease-modifying anti-rheumatic drug (DMARD). Moreover, patients needed to have a wash out period of at least one month from the last TNF blocker and at least six months follow up. All patients provided written informed consent and both participating clinics received approval by the local medical ethics committee. Demographic data and clinical characteristics are presented in Table 1.

**Table 1:** Baseline characteristics and response-status in RTX treated RA patients.

	Test cohort (*n *= 14)	Validation cohort (*n *= 26)	Total group (*n *= 40)
**Demographics**			
Age, years	55 ± 10	59 ± 11	58 ± 11
Female, %	86	85	85
**Disease characteristics**			
RA duration, years	11 (5 to 21)	6 (2 to 16)	8 (3 to 17)
DAS28-score	5.7 ± 1.0	5.8 ± 1.2	5.8 ± 1.2
ESR, mm/hr	14 (9 to 31)	29 (13 to 50)	23 (12 to 42)
CRP, mg/L	9 (3 to 19)	14 (6 to 27)	12 (4 to 25)
IgM RF positive, %	71	63	68
ACPA positive, %	79	70	73
Erosive diseases, %	93	59	72
**Medication**			
Previous biologicals, n	2 (1 - 2)	2 (1 - 2)	2 (1 - 2)
> 1 Biological in history, %	50	50	50
Previous DMARDS, n	4 (4 - 5)	3 (3 - 4)	4 (3 - 4)
Current prednisolone use, %	93	63	70
Prednisolone dosage, mg/day	9 (7 - 13)	5 (0 - 10)	8 (0 - 10)
Current DMARD use, %	93	77	83
Current statin use, %	21	13	18
**Response**			
ΔDAS28	-1.6 (-2.7 - -0.5)	-1.0 (-1.5 - 0.2)	-1.0 (-2.0 - 0.3)
ΔDAS28 > 1.2, % EULAR responders, %	57 64	65 50	63 55

Continuous variables are presented as mean with standard deviation or median with interquartile range where appropriate. ACPA, anti-cyclic citrullinated protein; CRP, C-reactive protein; DMARD, disease modifying anti-rheumatic drug; ESR, erythrocyte sedimentation rate; HCQ, hydroxychloroquine; MTX, methotrexate; RF, rheumatoid factor; SSZ, sulphasalazyne;

### Treatment and clinical evaluation

Patients received RTX 1,000 mg intravenously on days one and 15 in combination with clemastine (2 mg intravenously), methylprednisolone (100 mg intravenously) and acetaminophen (1,000 mg orally), as premedication. Four weeks after the first infusion and from 12 weeks on every three months, the DAS28 was assessed for disease activity status [19]. After six months the presence of a clinical nonresponse was defined in two ways: as ΔDAS28 < 1.2, and as EULAR nonresponse (additionally requires DAS28 to be > 5.1) [20]. At these visits, serum and whole blood samples for RNA analysis were collected using PAXgene tubes (PreAnalytix, GmbH, Hilden, Germany. The use of concomitant DMARDs, prednisolone or NSAIDs during the study duration was permitted. Retreatment with RTX or treatment with other biological agents was prohibited during the six-month follow up.

### Laboratory Measurements

#### RNA isolation

For RNA isolation, 2.5 ml blood was drawn in PAXgene tubes, incubated for two hours at room temperature and stored at -20°C. Tubes were thawed overnight at room temperature prior to RNA isolation. Total RNA was isolated using Bio robot MDX (Qiagen, Benelux b.v., Venlo, The Netherlands) according to the manufacturer's instructions (PAXgene Blood RNA Mdx kit). Samples were cleaned from salts that may be present using the Qiagen RNA MinElute procedure according to the manufacturer's procedure (Qiagen, Venlo, The Netherlands). Total RNA concentration was measured using the Nanodrop spectrophotometer (Nanodrop Technologies, Wilmington, DE, USA) and RNA purity and integrity was verified using lab-on-chip technology (Agilent 2100 Bioanalyzer, Santa Clara, CA, USA).

#### Microarray analysis

Genome-wide transcriptome data were collected from peripheral blood cells of 14 patients prior to the start of RTX using baseline transcript data from 13 patients generated earlier for analysis of the pharmacological changes during RTX [21], supplemented with baseline data from an additional patient. Transcriptome data were generated using the HumanHT-12 v3 Expression BeadChip (Illumina, San Diego, CA. USA). In this process, 500 ng total RNA was used to synthesize biotin-labeled cRNA using the Illumina^® ^TotalPrep™ RNA amplification kit (Ambion, Austin, TX, USA) and 750 ng biotinylated cRNA was hybridized onto the Illumina beadchip (ServiceXS, Leiden, the Netherlands). Bead summary intensities were log2-transformed and normalized using quantile normalization [22,23]. All microarray data have been submitted to the Gene Expression Omnibus (GEO) under accession number GSE37107.

Hierarchical cluster analysis was used to categorize the data and TreeView was used to visualize differentially expressed genes [24]. Baseline characteristics of RA patients were expressed as mean (SD) or median (interquartile range (IQR)), where appropriate.

#### cDNA synthesis and quantitative real time PCR

Quantitative real-time PCR was performed on total RNA samples from the patients in the validation group (*n *= 26) using an ABI Prism 7900HT Sequence detection system (Applied Biosystems, Foster City, CA, USA). RNA (0.5 μg) was reverse transcribed into cDNA using a RevertAid H-minus cDNA synthesis kit (MBI Fermentas, St. Leon-Rot, Germany) according to the manufacturer's instructions. Gene expression levels of one housekeeping gene, glyceraldehyde-3-phosphate dehydrogenase (*GAPDH*), and eight IRGs, that is, *LY6E*, *HERC5, IFI44L, ISG15, MxA, MxB, EPSTI1 *and *RSAD2*, were determined. To calculate arbitrary values of mRNA levels and to correct for differences in primer efficiencies for each gene a standard curve was constructed. Expression levels of target genes were expressed relative to *GAPDH*.

### Calculation of interferon (IFN) score

An IFN score was calculated as an average of expression levels of eight IRGs, namely, *Ly6E, HERC5, IFI44L, ISG15, MxA, MxB, EPSTI1 *and *RSAD2*, log2 based. Where indicated another set of IRGs was used. For univariate analysis and bivariate logistic regression analysis the IRG response data from both the test and validation cohort were median centered (as log2 transformed data) and combined to calculate the IFN score for further analysis.

### Statistical analysis

Statistical analysis on microarray data was performed using Significance Analysis of Microarray (SAM), version 3.09 [25]. Two class paired analyses using SAM at a false discovery rate (FDR) of less than 5% between them were applied to search for single genes that were significantly differentially expressed between responders and non-responders upon RTX treatment. Cluster analysis was used for the categorization of coordinately differentially expressed genes [24]. TreeView was used to visualize differentially expressed genes [26]. Ingenuity pathway analysis [27] was used for pathway level analysis.

Differences in gene expression levels of IFN type I network genes between patients with a ΔDAS28 > 1.2 versus ΔDAS28 < 1.2 or between EULAR good/moderate versus non-responders were analyzed using the Student unpaired t test.

To select the best performing gene set of the eight IRGs for the ROC-curve performance we calculated the average expression of all combinations using R [28]. ROC curves were then constructed by applying the ROC package [29] available in Bioconductor. Responder status was based on the ΔDAS28 non-responder status. The Area under the Curve (AUC) was calculated for each curve using the AUCi (integrated) function.

Stepwise regression analyses were performed to find associations between IRG gene expression levels and clinical response. Firstly, crude odds ratios (ORs) with 95% confidence intervals (CI) were calculated. Due to the number of patients included, it was not possible to perform multiple logistic regression analysis. Instead, the association between potential predictors and clinical outcome based on ORs with 95% CIs was compared to that for the IRG set. Subsequently, potential predictors for RTX response were added as covariates in a stepwise bivariate logistic analysis, that is, 1) seropositivity for IgM RF or ACPA, 2) lower number of previous TNF blocking agents, 3) use of lipid lowering drugs, 4) presence of erosions, 5) use of prednisolone, and 6) use of methotrexate.

All regression analyses were performed using SPSS version 15.0 (SPSS, Chicago, IL, USA).

*P *values < 0.05 are considered to be significant.

## Results

### Patient characteristics

A total of 40 patients were included, 14 of whom were subjected to genome-wide gene expression profiling (test cohort) and 26 of whom were used for validation (validation cohort). The clinical characteristics of these patients are shown in Table 1. Concomitant prednisolone use was lower in the validation cohort, although the daily dosage was not significantly different. Other characteristics were not significantly different between the test and validation cohort.

### Interferon Response Genes discriminate between non responders and responders

In order to identify molecular markers that discriminate responders from non-responders to RTX in RA we analyzed the whole blood cell gene expression profile of 14 RA patients before the start of RTX treatment. Therefore, we searched for genes and/or gene signatures that were differentially expressed between responders (eight patients with a ΔDAS28 > 1.2 after six months) and non-responders (six patients with a ΔDAS28 ≤ 1.2). Significant Analysis of Microarrays (SAM) at a FDR of less than 5% did not reveal differentially expressed genes at the single gene level. Subsequent analysis based on fold differences identified a total of 124 genes which differed at least two-fold in expression level (compared to the median expression level) in at least three out of the 14 patients. Supervised hierarchical cluster analysis, wherein patients were categorized as responders and non-responders based on ΔDAS28, categorized these 124 genes into five groups, which represented different biological themes. Cluster one consists of genes from the IFN type I network which are related to IRF (IFN Regulatory Factor)-activation and IFN type I response activity. Genes of cluster two belong to processes such as DNA replication, recombination, and repair. Cluster three represents genes involved in protein synthesis, and cell-to-cell signaling and interaction. Genes involved in hematological system development and function and the immune cell trafficking network were characteristic for cluster four. Cluster five consists of genes mainly involved in immune regulation, such as NFAT regulation, complement system, B-cell development and the antigen presentation pathway

**Figure 1:**
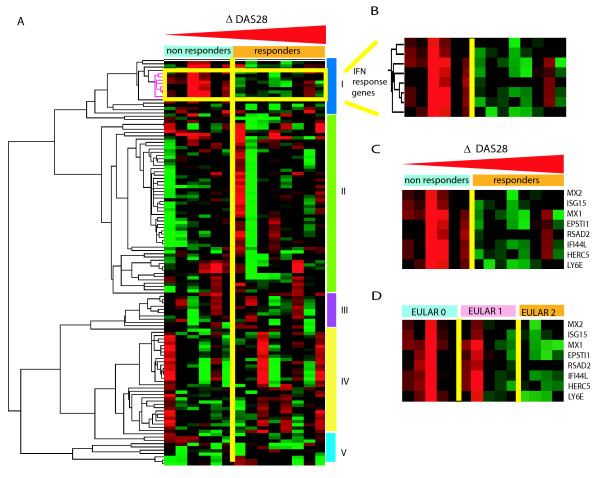
**Cluster diagrams of genes that were differentially expressed between 14 patients with RA before the start of rituximab treatment in relation to the ΔDAS28 response outcome at six months**. **A**. Supervised (one-way) hierarchical cluster analysis of baseline gene expression levels of a set of 124 genes that differed at least two-fold in at least three patients. Genes (rows) that are increased relative to the mean are indicated in red, decreased in green and genes that show no difference are indicated in black. Patients were stratified based on changes in ΔDAS28 at six months after the start of treatment in responders (indicated by the orange bar) and non-responders (indicated by light blue bar) ('change in ΔDAS28' from low (left) to high (right)). The supervised analysis revealed five gene clusters of which one, consisting of IRGs, was associated with clinical outcome**. B**. An expanded view of the subcluster of eight IRGs that is associated with clinical responder status. **C**. Cluster of eight IRGs associated with ΔDAS28 clinical responder status. **D**. Cluster of 8 IRGs associated with EULAR clinical responder status. DAS28, 28 joints disease activity score; EULAR, European League against Rheumatism; IRG, interferon response genes.

**Figure 2:**
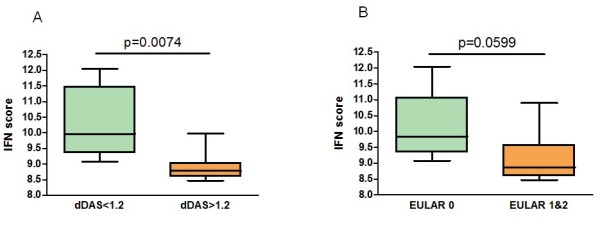
**Differential expression of type I interferon (IFN)-response gene activity in relation to clinical responder status to RTX**. The expression levels of eight IRGs (*LY6E, HERC5, IFI44L, ISG15, MxA, MxB, EPSTI1 *and *RSAD2) *were determined by quantitative (q)PCR analysis in peripheral blood cells of patients with RA before RTX treatment. Average expression in Log2 of the eight IRGs was calculated and used as IFN-score. Data are shown as box plots; each box shows the 25^th ^to 75^th ^percentiles. Student t test analysis revealed a significantly higher IFN-score in non-responders compared to responders based on ΔDAS28 (*P *= 0.0074) (**A**), and EULAR good/intermediate responders (1, 2) versus non-responders (0) (*P *= 0.0599) (**B**). PCR, polymerase chain reaction.

Next, we analyzed whether clusters of coordinately regulated genes are associated with clinical outcome (Figure 1A). The average expression levels of the genes per cluster were calculated and related to the clinical ΔDAS28 response status at six months following the start of RTX treatment. This analysis revealed a significant differential expression only of cluster one, consisting of IFN-type I response genes (IRGs). This cluster was represented by eight type I IRGs, namely, *LY6E, HERC5, IFI44L, ISG15, MxA, MxB, EPSTI1 *and *RSAD2*, whose expression levels were highly correlated (r = 0.91) (Figure 1B, C and 1D). A low expression of IRGs was associated with a good clinical response, whereas patients with increased expression of these genes exhibited a poor response (*P *= 0.0074) (Figure 1C and 2A). Comparable results were observed when response status was assessed by the EULAR response criteria in good/intermediate responders versus non-responders, although this association did not reach statistical significance (*P *= 0.0599) (Figure 1D and 2B).

### Prediction of response to RTX

**Figure 3:**
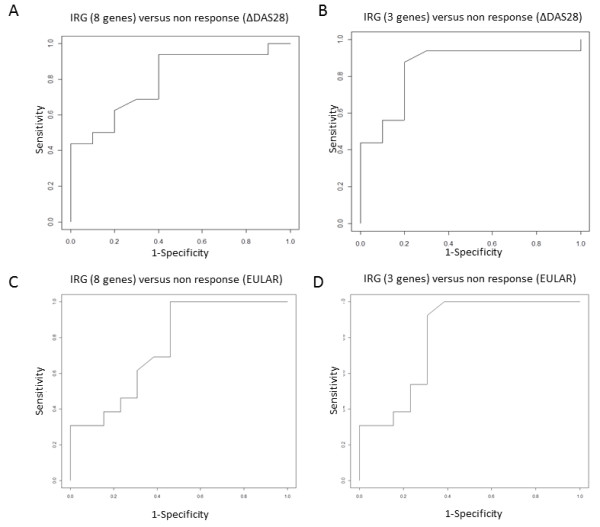
**Receiver operating characteristics (ROC) curves for the IRGs as predictor for nonresponse upon RTX treatment in the validation cohort (*n *= 26)**. **A**. AUC (0.82) for the eight IRG set based on ΔDAS28 response criteria, **B**. AUC (0.87) for the three IRG set based on ΔDAS28 response criteria, **C**. AUC (0.78) for the eight IRG set based on EULAR response criteria (responders and intermediate responders vs. non-responders) and **D**. AUC (0.83) for the three IRG set based on EULAR response criteria (responders and intermediate responders versus non-responders). On the y-axis sensitivity and on the x-axis 1-specificity is indicated. AUC, area under the curve; DAS28, 28 joints disease activity score; EULAR, European League against Rheumatism; IRGs, interferon response genes; RTX, rituximab.

An important measure of the accuracy of the eight IRG-set in separating the responders and non-responders is the ROC curve AUC analysis. Here we used the average expression values of the eight IRGs as an IFN score to construct an ROC curve using an independent cohort of 26 patients and calculated the AUC, which was evaluated as a predictor for non-response. At six months after the start of RTX treatment nine patients were classified as ΔDAS28 responders and 17 as non-responders. This analysis revealed an AUC of 0.82 according to the six-month ΔDAS28 criteria (Figure 3A).

In order to select the most optimal gene set for response prediction based on ROC-curve AUC criteria we tested all combinations of the eight IRGs. For each combination, the average expression was calculated per patient and ROC curves were then constructed by applying the ROC package available in Bioconductor [29]. Responder status was based on the ΔDAS28 non-responder status. Combinations of three, four or six specific IRGs (*EPSTI1, RSAD2, MxA; or HERC5, RSAD2, MxA, LY6E; or HERC5, IFI44L, EPSTI1, RSAD2, MxA, LY6E*, respectively) revealed the highest AUC of 0.87 (Figure 3B).

For both the eight IRG and three IRG sets we also calculated the ROC-curve AUC for the EULAR response outcome. These analyses revealed an AUC of 0.78 and 0.83, respectively, demonstrating that these sets also have clinical utility for response prediction based on the EULAR response criteria (Figure 3C and 3D).

Next, we studied the strength of the association between the eight IRG-set and non-response in the context of other potential predictors. We combined the data from the test and validation cohort. Univariate analysis showed that the eight gene sets were negatively associated with clinical response defined as ΔDAS28 < 1.2 (OR: 0.25, 95% C.I.: 0.09 to 0.70, *P *= 0.008, Nagelkerke R^2^0.379). Similar findings were made for the three gene set (OR: 0.25, 95% C.I.: 0.10 to 0.65, *P *= 0.004, Nagelkerke R^2^0.42). The IRG sets outperform the association of other factors such as: 1) seropositivity for IgM RF or ACPA (OR: 1.6, 95% C.I.: 0.92 to 2.83, *P *= 0.097); 2) lower number of previous TNF blocking agents (OR: 0.29, 95% C.I.: 0.077 to 1.06, *P *= 0.061); 3) use of lipid lowering drugs (OR:1.8, 95% C.I.: 0.35 to 9.41, *P *= 0.481); 4) presence of erosions (OR:0.63, 95% C.I.: 0.15 to 2.54, *P *= 0.511); 5) use of prednisolone (OR:2.0, 95% C.I.: 0.49 to 8.20, *P *= 0.335); and 6) use of MTX (OR: 0.3, 95% C.I.: 0.076 to 1.15, *P *= 0.078). To determine if the IRG sets are predictors of clinical outcome independently from the other potential predictors, stepwise bivariate analysis was performed. These analyses revealed that adjustment for each single variate, that is, 1) seropositivity for IgM RF or ACPA; 2) more than one previous TNF blocking agent; 3) use of lipid lowering drugs; 4) presence of erosions; 5) prednisolone use; and 6) MTX use as well as age and gender had no significant influence on the association between IRGs (the eight or three gene set) and ΔDAS28.

## Discussion

B-cell depletion with the anti-CD20 antibody RTX has proven efficacious in RA [7,8,30]. However, clinical experience has revealed that approximately 40% to 50% of the patients show no clinical response. Genome-wide transcript profiling technology provides a very powerful and robust tool allowing an open-ended survey to identify comprehensively the fraction of genes that are differentially expressed between responders and non-responders before the start of treatment. This approach identified a selective group of genes that are all regulated by type I IFN that is significantly associated with clinical response. Our results not only confirm but also extend preliminary results reported previously [17]. In that study a candidate-gene approach was used to demonstrate an association between the expression of three randomly picked IRGs (*Mx1, ISG15 *and *OAS1) *by peripheral blood mononuclear cells (PBMC) and clinical outcome of RTX treatment. Here, we further extend this initial observation. Firstly, we demonstrated that out of all the genes in the human genome only the IRGs are associated with clinical response to RTX. We identified a set of eight IRGs whose expression status could be translated into a score that showed a significant correlation with response outcome. Secondly, we demonstrated clinical utility by showing that the eight gene-based IFN score predicts the response to RTX using ROC-curve analysis with an AUC of 0.82 in an independent validation cohort. Lastly, advanced data analysis identified a subset of three gene markers that more accurately and robustly predicts the response to RTX therapy (AUC 0.87). For the future, larger and prospective multicenter studies are needed to further validate these findings.

The ROC AUC is an important measure of test accuracy. For the eight gene set assay an AUC of 0.82 was reached, which means that this test correctly classifies 82% of two patients of randomly drawn pairs, which is considered 'good' [31]. Based on these data a cut-off could be chosen to predict non-response to RTX with a specificity of 100% and a sensitivity of 44%. Advanced classifier analysis yielded an AUC of 0.87 for a three gene test, which is considered close to excellent (AUC > 0.90) at separating responders from non-responders. The association of IRG and RTX outcome appeared independent of other proclaimed biomarkers for RTX treatment. Comparable findings were generated based on the EULAR response outcome measure.

In our study clinical nonresponse at six months was defined by two composite scoring indices: 1) as ΔDAS28 < 1.2 and 2) as EULAR non-response (additionally requires DAS28 to be > 5.1). The use of a composite scoring index could negatively impact the overall response score since individual components (such as joint swelling and tenderness) could be the consequence of irreversible damage in patients who have failed TNF-blocking therapy, who are the ones that are treated with RTX. Moreover, a limitation of our study is that we analyzed the predictive value of IRG for RTX response only at six months after initiation of RTX. Although this time point is in line with the time point of the primary outcome of previous randomized controlled trials in RTX-treated RA patients [8,9], we would like to evaluate other time-points as well.

Several investigators have reported on an increased expression of IRGs in peripheral blood cells in a subset of RA patients [17,21,32-35]. Evidence for a role of IFNα and IFNβ in the induction of IFN response activity in RA was provided by Mavragani and colleagues [33] who demonstrated the concomitant presence of IFN bioactivity, which could be inhibited by neutralizing antibodies against IFNα and IFNβ, in the serum of RA patients. Type I IFNs have an essential function in mediating innate immune responses against viruses and play critical roles in several immunological processes including lymphoid differentiation, homeostasis, tolerance and memory. Although an increased expression of IRGs was associated with the persistence of ACPA after TNF blockade, a direct relationship between IRG expression and autoantibody responses in RA could not be confirmed [36]. Accordingly, we found that the relationship between the IRG activity and response outcome was independent of auto-antibody status.

Results from our study suggest that IFN^high ^RA patients represent a different pathogenic subset of RA marked by a failure to respond to B-cell depletion therapy. A simple explanation could be that the pathogenesis in IFN^high ^patients is less dependent on B-cells, compared to IFN^low ^patients. However, the IRG activity was found to be equally present in seropositive and seronegative RA patients, arguing against an association between IFN-response activity and pathogenic B cells [36]. Alternatively, a high baseline IFN-activity may be associated with the presence of a subset of pathogenic B cells insensitive to the effects of RTX. These could be present at baseline and could survive in synovial or bone marrow tissues due to, for example, incomplete B-cell depletion effectors or concomitant expression of B-cell survival factors, such as BAFF/BLyS [37]. IFNs may also affect B-cell differentiation, such as *in situ *differentiation in CD20^- ^plasma blasts [38].

Pharmacological studies revealed that the regulation of IRG-activity during treatment is associated with the IFN-response activity prior to the start of treatment [21]. The IRG-activity in non-responders, who have an increased IRG-activity before the start of treatment, remains stable during treatment, whereas good responders, who have a low or absent IFN-response activity at baseline, develop IFN-response activity till a level comparable to that of non-responders during three months of treatment. Thus, baseline IRG-activity appears to be crucial for the pharmacological induction of type I IFN-response activity, which might be a critical event in the ameliorative effect of B-cell depletion therapy in RA. The increased IFN-activity might explain the increased BAFF/BLyS levels and persistence of pathogenic B cells and could explain the change in macrophage function reflected by an increased BAFF/BLyS, IL10 and CD86 mRNA expression [39].

## Conclusions

In conclusion, we applied genome-wide gene expression technology to identify a set of markers, which accurately and robustly predict the response to RTX treatment in an independent validation group of RA patients. Therefore, these findings are likely to become a substantial aid to the physician, taking the paradigm of personalized medicine one step further.

## List of abbreviations

ACPA: anti-cyclic citrullinated proteins antibodies; AUC: area under curve; CI: confidence interval; CRP: C reactive protein; DAS28: 28 joints disease activity score; DMARD: disease modifying anti-rheumatic drug; ESR: erythrocyte sedimentation rate; EULAR: European League against Rheumatism; HCQ: hydroxychloroquine; IFN: interferon; IgM: immunoglobuline M; IQR: interquartile range; IRG: interferon response genes; MTX: methotrexate; NSAID: non steroidal anti-inflammatory drug; OR: odds ratio; RA: rheumatoid arthritis; RF: rheumatoid factor; ROC: receiver operating curve; RTX: rituximab; SD: standard deviation; SSZ: sulphasalazyne; TNF: Tumor necrosis factor.

## Competing interests

Dr Nurmohamed has received research and speaking fees from Roche

Prof. Lems is member of advisory board van Roche, Abbott and MSD.

Prof. Dijkmans has received research grants from Wyeth, Abbott and Schering-Plough.

Prof. Verweij is an inventor on a patent application on the predictive value of IFN-type I response activity for the clinical outcome of B-cell depletion therapy. Prof. Boers has received consultation fees of more than $10,000 from Roche and less than $10,000 from Glaxo Smith Kline, Novartis, AstraZeneca, Abbott, UCB, Schering-Plough and Bristoll-Myers Squibb

Mr. Raterman MD, Mr. S. de Ridder MSc, Mrs. Vosslamber MSc, Dr. van der Wiel and Dr. Voskuyl have no competing interests to declare.

## Authors' contributions

HR was responsible for the acquisition of data, analysis and interpretation of data and drafting of the manuscript. SV performed the RNA experiments, analyzed and interpreted the data and drafted the manuscript. MN, WL, MB, MvdW, SdR, and BD assisted with analysis and interpretation of data and revised the manuscript critically. CV and AV conceived and designed the study, provided technical supervision, analysis and interpretation of the data and revised the manuscript critically. All authors read and approved the final manuscript
